# Impacts of soil nutrition on floral traits, pollinator attraction, and fitness in cucumbers (*Cucumis sativus* L.)

**DOI:** 10.1038/s41598-022-26164-4

**Published:** 2022-12-16

**Authors:** Anthony D. Vaudo, Emily Erickson, Harland M. Patch, Christina M. Grozinger, Junpeng Mu

**Affiliations:** 1 Department of Biology, University of Nevada Reno, Reno, NV 89557 USA; 2 Department of Entomology, Center for Pollinator Research, Huck Institutes of the Life Sciences, The Pennsylvania State University, University Park, PA 16802 USA; 3 Ecological Security and Protection Key Laboratory of Sichuan Province, Mianyang Normal University, Mianyang, 621000 China

**Keywords:** Ecology, Plant sciences

## Abstract

Annual plants allocate soil nutrients to floral display and pollinator rewards to ensure pollination success in a single season. Nitrogen and phosphorus are critical soil nutrients whose levels are altered by intensive land use that may affect plants’ fitness via pollinator attractiveness through floral display and rewards. In a controlled greenhouse study, we studied in cucumbers (*Cucumis sativus*) how changes in soil nitrogen and phosphorus influence floral traits, including nectar and pollen reward composition. We evaluated how these traits affect bumble bee (*Bombus impatiens*, an important cucumber pollinator) visitation and ultimately fruit yield. While increasing nitrogen and phosphorus increased growth and floral display, excess nitrogen created an asymptotic or negative effect, which was mitigated by increasing phosphorus. Male floral traits exhibited higher plasticity in responses to changes in soil nutrients than female flowers. At 4:1 nitrogen:phosphorus ratios, male flowers presented increased nectar volume and pollen number resulting in increased bumble bee visitation. Interestingly, other pollinator rewards remained consistent across all soil treatments: male and female nectar sugar composition, female nectar volume, and pollen protein and lipid concentrations. Therefore, although cucumber pollination success was buffered in conditions of nutrient stress, highly skewed nitrogen:phosphorus soil ratios reduced plant fitness via reduced numbers of flowers and reward quantity, pollinator attraction, and ultimately yield.

## Introduction

Annual plants allocate the nutrients they obtain from soil to vegetative growth and reproduction, including seed and fruit development within a single season^1^. Because nearly 80% of flowering plants benefit from animal-mediated pollination for reproduction^2^, most annual flowering plant species need to invest soil nutrients to increase attractiveness to potential pollinators and ensure visitation and pollination success through floral cues (e.g. display and scent) and rewards of nectar and pollen^2^. Nitrogen and phosphorus are well-studied soil nutrients contributing to vegetative growth. Increases in either nutrient results in faster plant growth^3–5^. Nitrogen and phosphorus have also been shown to influence floral traits, increasing the number and size of flowers^6–9^, pollen number and size^6,10–12^, nectar secretion rates^7,8,13,14^ and sugar ratios^9^. Importantly, landscape levels of nitrogen and phosphorus can be profoundly influenced by anthropogenic change^15^. However, little research exists examining the effect of soil nutrients on floral cues and reward, pollinator attraction, and ultimately plant reproduction^16^ (yet see^7,8,17–19^). Using cucumbers (*Cucumis sativus* L.) and bumble bees (*Bombus impatiens* C.) as a model system, we examine the influence of nitrogen and phosphorus on a suite of floral traits and how these traits influence pollinator visitation rates and plant reproductive success through fruit production.

Nectar and pollen rewards are essential for pollinator health and plant reproduction. Nectar is the primary source of carbohydrates for most pollinators^20^, and pollen is the main source of dietary protein and lipids^21^. Nectar and pollen quality (nectar sugar concentrations and composition; pollen protein and lipid concentrations), and quantity (nectar volume, pollen number per flower, and number of flowers) are therefore important to ensure pollinator attraction and foraging fidelity to plants and thus promote conspecific pollen transfer. For instance, bumble bees, which are key pollinators of wild and agricultural plants, are more attracted to higher nectar concentrations and volumes^22^, and high protein:lipid ratios of pollen among different plant species^23,24^. These floral reward traits are influenced by nitrogen and phosphorus soil nutrients^8,9,13,14,25,26^, and variation in these traits can affect plant attractiveness to pollinators^25–28^, and even pollinator health^25,26,29–32^. Most research, however, has not directly integrated or linked impacts of soil nutrients on floral reward nutritional chemistry to pollinator visitation, and plant reproduction and fitness. Establishing how these factors and traits interact is essential for understanding plant reproductive biology in response to variation in environmental conditions^16^.

Plants are sessile and therefore constrained by soil nutrient availability. For annual plants, this requires partitioning available nutrients to maximize reproductive output within a single growing season^1,33^, which may result in selection for strategies to compensate for nutrient limitation or surplus. Thus, plants may prioritize certain floral traits to ensure consistent and stable pollinator visitation across variable soil nutritional contexts. For instance, nectar sugar and pollen nitrogen content have been found to be consistent in the face of fertilizer or nitrogen enrichment^7,8^. However, anthropogenic changes to the landscape have caused alteration of soil nutrient profiles that may have exceeded plants’ ability to buffer against variations in soil nutrition. For example, land use can reduce soil nutrient availability through soil nutrient runoff, reduction in soil fertility, and pollution^15,16^, and thereby *reducing* plants’ ability to grow, flower, and attract pollinators and reproduce. On the other hand, excessive soil enrichment (e.g. soil eutrophication), occurs from fertilization and accumulation of nutrients from runoff^15,16^. These smaller increases in soil nutrients may, in the short term, *increase* floral attractiveness in plants^8,17^. However excess nitrogen in soil has been considered a threat to biodiversity at a landscape level^16,34^. Excess soil nitrogen can result in unbalancing nitrogen and phosphorus ratios^35,36^, and cause nitrogen toxicity^37^, potentially harming essential plant reproductive traits which may result in detrimental effects on pollinator attraction and health^8,26,38^. These conditions of soil nutritional limitation and surplus could therefore have overall detrimental effects to an individual plant’s fitness. In this study, we examine both how annual plants respond to soil nutrient stress and how they may exhibit pollination resilience in the face of this stress.

Here, we present one of the first studies examining the influence and interaction of nitrogen and phosphorus soil nutrients on a plant's floral traits, including reward nutritional quality, and directly testing these effects on pollinator attractiveness and plant fitness^16,18,19,26^. We studied the plant’s complete life cycle and measured the effects of nitrogen and phosphorus nutrient concentrations and ratios ranging from limitation to surplus. We used cucumbers (*C. sativus*) as our model system; these are annual monoecious plants, where separate male and female flowers grow on the same plant. Male flowers produce pollen and nectar, while female flowers produce nectar rewards for pollinators, and require pollen transfer from male to female flowers to fertilize and set fruit (except for some commercial varieties). This allowed us to separately examine traits associated with both male and female flowers on the same plant. We show how variation in soil nitrogen and phosphorus affect vegetative growth, floral display and rewards including pollen and nectar quality and quantity, and how changes in these traits influence pollinator (bumble bee, *B. impatiens*) visitation and plant reproductive output. We hypothesize that, within certain parameters, increased nitrogen and phosphorus will increase floral traits attractive to pollinators, but nutritional stress through limitation or excess negatively affects floral traits and pollinator attraction.

## Results

### Evaluation of impact of soil nutrients through regression analysis

For our analyses, we included a 0:0 ppm nitrogen phosphorus treatment (nitrogen:phosphorus ratio of 1:1) where no fertilizer was added to the plants. We examined the effect of increasing nitrogen concentrations from when phosphorus concentrations were held constant (50:25, 100:25, 200:25, or nitrogen:phosphorus ratios of 2:1, 4:1, 8:1). We also included a 200:50 ppm nitrogen:phosphorus treatment (at a ratio of 4:1), where we could evaluate the impact of increasing phosphorus further.

**Figure 1:**
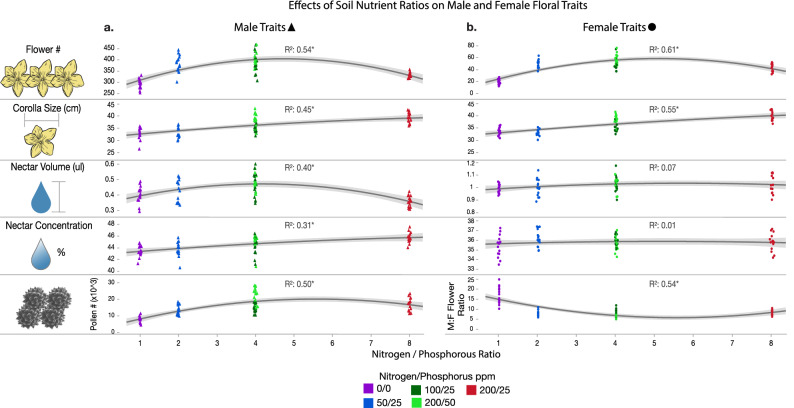
The effects of nitrogen:phosphorus soil nutrient ratios on floral traits (Supplementary Table S3). Regression lines are fitted with the quadratic nutrient ratio terms. Each marker represents a different individual plant, with each marker colored by their treatment group and shaped by male or female flowers. Nitrogen ppm/phosphorus ppm of nutrient solutions for each treatment group are labeled in the legend. Note that male floral traits frequently improve as ratios increase to 4:1, yet reach asymptotic or decreased levels at 8:1. Similar effects are noticed for female traits. However neither female nectar volume nor concentration were associated with changes in soil nutrients. The effects of nitrogen and phosphorus individually are provided in Fig. 5, Fig. S2, and Supplementary Table S2.

**Figure 2:**
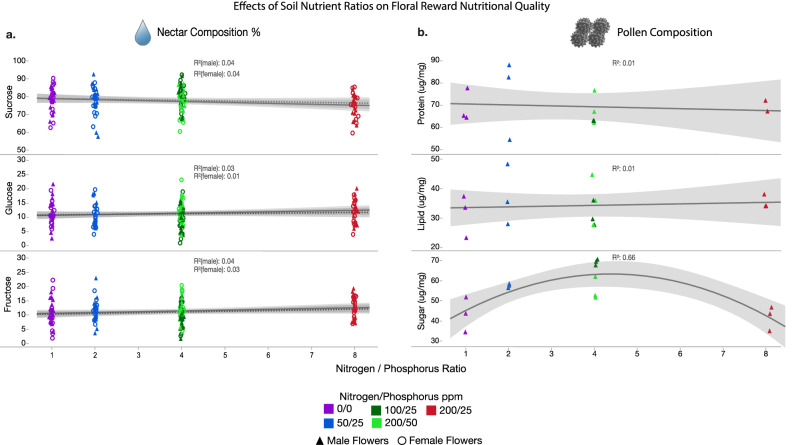
The effects of nitrogen:phosphorus soil nutrient ratios on floral reward composition (Supplementary Table S3). Regression lines are fitted with the quadratic nutrient ratio terms. For nectar, each marker represents a different individual plant. For pollen, each marker represents a pooled replicate. Each marker is colored by their treatment group and shaped by male or female flowers. Nitrogen ppm/phosphorus ppm of nutrient solutions for each treatment group are labeled in the legend. Note that changes in soil nutrient ratios did not affect male or female nectar sugar composition, nor pollen protein concentrations, lipid concentrations, or P:L ratios. Note that pollen sugar concentrations improved as ratios increased to 4:1, yet decreased at 8:1 (pollen sugar concentrations had no effect on bee visitation rates). The effects of nitrogen and phosphorus individually are provided in Supplementary Table S2.

We examined using multiple regression 1) the effects of nitrogen and phosphorus ppm and their interaction on vegetative traits, floral traits, and floral reward composition traits (Supplementary Table S2). We then examined 2) the effects of nitrogen:phosphorus ratios on vegetative traits (Fig. S1, Supplementary Table S3), floral traits (Fig. 1; Supplementary Table S3), and floral reward composition traits (Fig. 2; Supplementary Table S3). We then examined with regression analysis the effects of floral traits in bumble bee visitation rate, then the effects of visitation rate on fruit yield.

In many analyses, increased nitrogen at the highest nitrogen:phosphorus ratio (8:1) had negative effects on different traits (Figs. 1, 2; Supplementary Tables S2,S3), that was mitigated by increased phosphorus at both 4:1 nitrogen:phosphorus ratios (200:50, 100:25). These trends are expressed in the negative effect of nitrogen ppm, or negative effect of nitrogen:phosphorus ratio quadratic terms in the regressions (Supplementary Tables S2,S3). This effect is also exemplified by the curved regression lines in Figs. S1, 1, 2. All significant regression results are reported below at *Adj R*^2^ ≥ 0.2 and *FDR P* < 0.05.

#### Vegetative traits

Soil nitrogen concentration was positively associated with plant height and above-ground biomass (Supplementary Table S2). Phosphorus had significantly positive effects by increasing above-ground biomass and reducing days to the first flower (Supplementary Table S2). However, as noted above, ratios of nitrogen to phosphorus exhibited the asymptotic or negative effect above 4:1 on plant height, above-ground dry mass, and days to first flower (Fig. S1; Supplementary Table S3).

#### Male floral traits

Nitrogen ppm was positively associated with flower size, nectar concentration, and pollen number, but negatively associated with flower number and nectar volume (Supplementary Table S2). Phosphorus ppm was strongly positively associated with male flower number, and positively associated with nectar volume (offsetting the effects of increased nitrogen; Supplementary Table S2); and strongly associated with pollen number per flower. As nitrogen:phosphorus ratio increased to 4:1, there was an increase in flower number, corolla width, nectar volume and pollen number, but there was a negative quadratic effect at 8:1 ratios, where these traits reached an asymptote or decreased (Fig. 1; Supplementary Table S3).

#### Female floral traits

Nitrogen ppm increased flower size but was negatively associated with the number of flowers (Supplementary Table S2). Phosphorus ppm on the other hand increased the number of flowers, and therefore decreased the ratio of male to female flowers, and was negatively associated with flower size (Supplementary Table S2). Similarly, nitrogen:phosphorus ratios again exemplified the positive but asymptotic effect, with maximum values at 4:1, on number of flowers, male:female flower ratio, and flower size (Fig. 1; Supplementary Table S3). Remarkably, and in contrast to male flowers, we did not detect any effect of soil nutrient ppm nor ratios on female nectar volume or concentration (Fig. 1; Supplementary Table S3).

#### Male reward composition

Neither nitrogen ppm, phosphorus ppm, and nitrogen:phosphorus ratios influenced pollen protein or lipid concentrations, P:L ratios, or sucrose, glucose, and fructose male nectar concentrations (Fig. 2; Supplementary Tables S2,S3). Pollen sugar concentration was positively affected by phosphorus ppm (Supplementary Table S2), and again negatively affected by the highest nitrogen:phosphorus ratio (Fig. 2; Supplementary Table S3). However, pollen sugar concentration had no effect on male flower visitation rate (*R*^2^ = 0.09, *P* = 0.57).

#### Female reward composition

Female nectar composition traits of relative sucrose, glucose, or fructose concentrations were not influenced by nitrogen ppm, phosphorus ppm, or nitrogen:phosphorus ratios (Fig. 2; Supplementary Tables S2,S3).

#### Pollinator visitation rate

**Figure 3:**
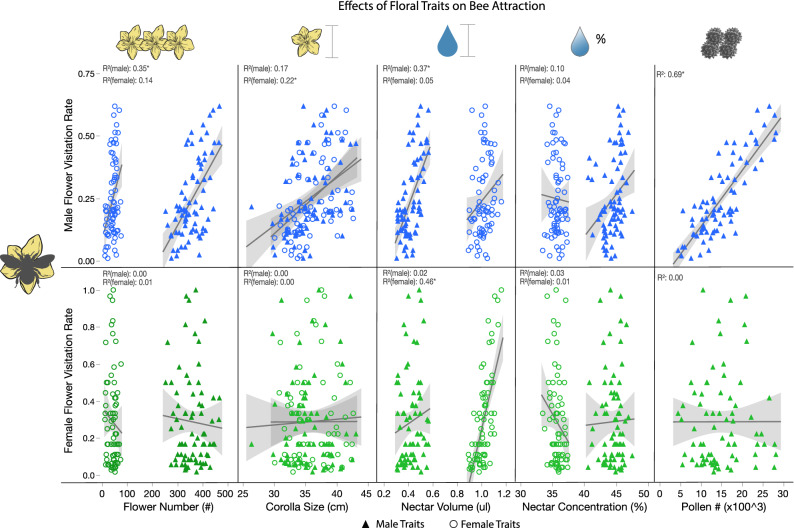
The effects of floral traits on bee visitation rates (Supplementary Table S4). The top and bottom rows represent male flower and female flower visitation rates respectively. Each marker is shaped by whether the predictor is a female flower trait (circle) or male flower trait (triangle). Note that male visitation rates are positively associated with many male flower traits, including flower number, size, volume, and pollen number. Yet, female flower visitation rate is only associated with female nectar volume.

*Male flowers:* The rate of bumble bee visitation to male flowers was positively linearly correlated to male flower number, male and female flower size, male nectar volume, male flower nectar concentration, and pollen number (Fig. 3; Supplementary Table S4). Interestingly, male flower visitation rate was negatively correlated to number of female flowers, perhaps because more female flowers (and lower male:female flower ratios) on these plants constituted some of the potential male visits. *Female flowers*: Pollinator visitation to female flowers was only linearly correlated to female nectar volume (which was not influenced by any nutrient concentration Fig. 3; Supplementary Tables S2, S3, S4).

#### Yield

**Figure 4:**
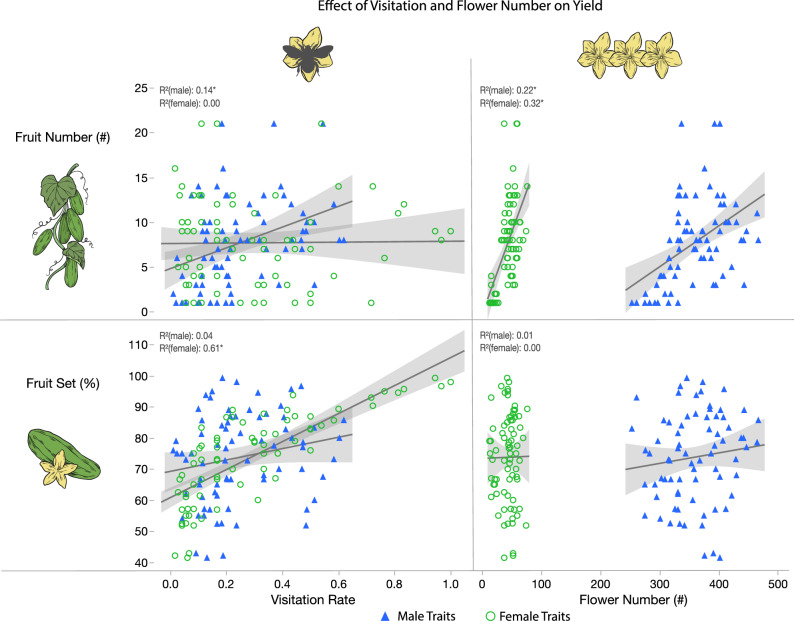
The effects of bee visitation rate and flower number on cucumber yield (Supplementary Table S5). Each marker represents an individual plant colored and shaped by male (blue triangles) or female (green circles) flowers. Note that fruit number was strongly influenced by flower number while fruit set (number of fruit per number of flowers per plant) was strongly influenced by female flower visitation rate.

The number of fruit per plant was positively related to male flower visitation rate (Fig. 4; Supplementary Table S5). Fruit set (proportion of female flowers producing fruit) increased with female flower visitation rate (Fig. 4; Supplementary Table S5), not flower number (Fig. 4). Fruit mass was not correlated to either male or female visitation rate (Supplementary Table S5), yet was strongly correlated to phosphorus ppm (Supplementary Table S2) and nitrogen:phosphorus ratios with the same quadratic trend as above (Supplementary Table S3).

### Evaluation of impact of soil nutrients through piecewise structural equation modeling

**Figure 5:**
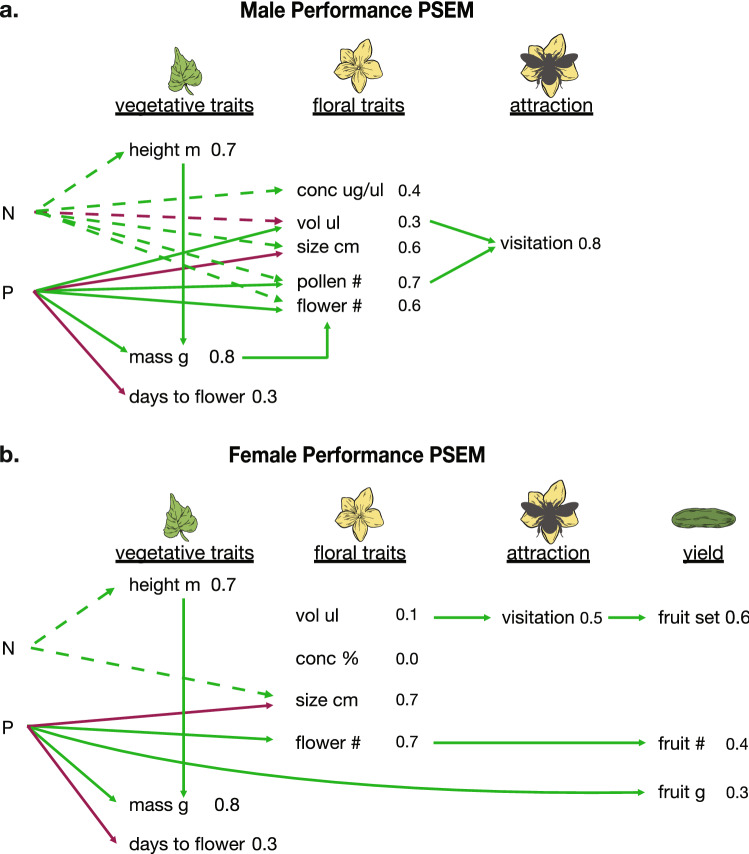
Piecewise SEM path analyses of soil nutrients on vegetative and floral traits, pollinator attraction/visitation, and reproduction/yield measures for male and female traits in cucumbers. Green lines represent significant positive relationships, whilst red lines represent significant negative relationships. Nonsignificant relationships are not represented for ease of path interpretation (full model paths and details are provided in Supplementary Tables S6, S7). *R*^2^ values for each linear regression response variable are provided. Estimated effects (including standardized estimates) for each variable are provided in Supplementary Tables S6, S7. Note how soil nutrients influence many male floral traits, yet nectar volume and pollen number were most strongly associated with male flower visitation rate, likely increasing male fitness. However, only female floral volume increased visitation rate, yet nectar volume was not influenced by changes in soil nutrient concentrations and remained consistent across treatments. Female fitness increased in three ways: (1) the path of increased soil phosphorus concentrations which in turn increased the number of female flowers, resulting in higher numbers of fruit; (2) the path of plants with higher female nectar volume having higher bee visitation rate and therefore a higher fruit set; (3) increased soil phosphorus concentrations resulting in larger fruit.

The above factors in the regressions were not completely independent of each other and therefore had potentially confounding variables. To mitigate this issue, we used piecewise structural equation modeling (PSEM^39^) to further examine the path of effects from soil nutrients on vegetative traits, to floral traits, to bee attraction and visitation, and finally reproduction or yield. It must be noted that the quadratic effects of nutrient ratios or individual nutrients could not be shown in PSEM models because they were restricted to only linear relationships^39^. We conducted PSEM analyses for male or female flower performance including both nitrogen and phosphorus as interacting or non-interacting independent variables. Full model details and results are presented in Supplementary Table S6 for male flowers, and Supplementary Table S7 for female flowers. Our most parsimonious and best explanatory models for male and female flower performance are presented in Fig. 5. Figure S2 includes PSEM data including the nitrogen × phosphorus interaction. Significance regression paths are reported below at *R*^2^ ≥ 0.3.

#### Male performance

The male performance PSEM was better explained *without* the interaction term (AIC = 215 vs. 228, Supplementary Table 6) where including nitrogen × phosphorus interaction did not improve the overall regressions’ *R*^2^ (Fig. 5a vs. S2; Supplementary Table 6). In the male PSEM, nitrogen and phosphorus ppm both significantly influenced all floral traits as explained above. But only male nectar volume (due to increasing phosphorus) and pollen number (due to increasing phosphorus and nitrogen) significantly influenced male flower visitation rate by bumble bees (Fig. 5a; Supplementary Table 6). Therefore, the plants with higher nutrients were able to produce more rewarding flowers, and in turn were more attractive to bumble bees, and likely increased their pollen dispersal (because of higher pollen number per flower and visitation rates).

#### Female performance

The female performance PSEM containing nitrogen × phosphorus interaction had a lower AIC value (298 vs. 307), but AICc improved greatly by the more parsimonious model *without* the interaction term (2071 vs. 4414; Fig. 5b vs. S2; Supplementary Table 7). For female performance, phosphorus was strongly associated with increased number of flowers but decreased flower size, while nitrogen ppm was more strongly positively influential on flower size. Yet flower number and size did not affect bee visitation rate. Bee visitation rate to female flowers was strongly influenced by higher nectar volume, yet determining what influenced nectar volume was elusive. The model with the interaction term shows several factors potentially affecting female nectar volume: while phosphorus was associated with increasing volume, other factors, including nitrogen × phosphorus, nectar concentration, and flower number, were negatively associated with volume (Fig. S2; Supplementary Table S7). However, none of these terms were significant in the model without the interaction term (Fig. 5b; Supplementary Table S7). As expected, visitation rate to female flowers increased the fruit set of plants, while the number of female flowers increased the number of fruits per plant. Soil phosphorus ppm increased fruit mass. Thus, while nutrients affected overall fruit production numbers via number of female flowers and size, visitation rate to female flowers remained consistent. Thus, our results demonstrate that female floral traits and rewards were held constant under a variety of soil conditions, buffering or ensuring pollination of all plants producing flowers.

## Discussion

In this study, we present a comprehensive analysis of how concentrations of soil nutrients nitrogen and phosphorus affect the lifecycle of annual monoecious (separate male and female flowers on the same individual plant) cucumbers through growth, pollinator attraction, and reproduction. We hypothesized that increased nitrogen and phosphorus will increase floral traits attractive to pollinators, but, outside of a certain range, nutritional stress through limitation or excess would negatively affect floral traits and pollinator attraction^8^. Using regression and structural equation modeling, we demonstrate that increasing soil nutrients generally increase vegetative traits and floral display, including flower number and size, as well as the floral rewards produced by male flowers (nectar concentration and volume, and amount of pollen), leading to increased attractiveness to pollinators and resulting in increased fruit production. However, very high soil nitrogen levels can have negative effects on several traits, and increasing phosphorus levels can mitigate some of these impacts. Intriguingly, some floral traits, mainly pollen nutritional quality, nectar sugar composition, and female flower nectar volume and concentration, remain consistent across soil treatments. Thus, these findings suggest that important floral rewards for pollinator attractiveness can be relatively constant to ensure pollination success, even in stressful conditions.

Importantly, while we show that soil nutrients can lead to particular positive outcomes of annual plants, increasing nitrogen alone can lead to toxicity of plants and increasing phosphorus may mitigate this problem, revealing ideal soil nutrient balances or ratios ^1,3,5,6,8,9,12,26,31^. Phosphorus tended to increase certain traits linearly from 0 to 50 ppm (Figs. 1, 2; Supplementary Table S2), while increasing nitrogen appears to asymptote or even reduce traits above 100 ppm (Figs. 1, 2; Supplementary Table S2). Therefore, the ideal soil nutrient nitrogen:phosphorus ratio tested was 4:1, which resulted in best overall growth, pollinator attraction, and reproduction (Figs. 1, 2; Supplementary Table S3).

We found that soil nutrients had differential effects on floral display and rewards between male and female flowers, which has interesting implications for plant reward systems and pollination ecology. Similar to previous studies on soil nutrients and floral traits, we found that increasing soil nutrients increased the number and size of cucumber male flowers per plant^6–9^, nectar concentration of male flowers^13,14^, and pollen number per flower^6,10–12^. Thus, under ideal nutrient conditions, plants may increase male fitness by producing more gametes (i.e. pollen), making it more attractive (e.g. larger floral display) and rewarding (nectar volume and pollen quantity) to bees, resulting in higher visitation (and possibly pollen dispersal) as we observed.

Pollen quality (protein and lipid concentrations, and protein:lipid ratios) remained the same under all soil conditions, revealing its importance as a physiological and ecological trait of male flowers. There could be two complementary reasons for this. First, pollen quality must remain at a particular level for the pollen’s own nutrient needs for reproduction, including keeping pollen cells alive, preventing desiccation, binding to receptive stigma, and germinating the pollen tube^40,41^. Thus, pollen quality may be an evolved trait non-negotiable for plants to ensure successful pollination and fertilization. But pollen also serves as a reward for bee visitation. Pollen protein:lipid ratio has been demonstrated to be important for bumble bee attraction and health^23,42–44^ and specific protein:lipid ratios may be important for attraction and the health of preferred pollinators^24^. Thus, maintaining pollen quality is necessary as an honest reward signal to bees.

Similar to male flowers, soil nutrients also influenced female flower size and number with more female flowers increasing fruit number^8^. Yet these traits did not statistically influence bee attraction and visitation, possibly because floral display was dominated by the sheer number of male flowers. All other aspects of female rewards did not change under any soil conditions tested, i.e. nectar volume, concentration, and sugar composition, which are important aspects of pollinator attraction for bumble bees who prefer higher concentrations and volumes^22^ and pollinators that may prefer certain sugar composition^21,45^. Nectar concentration and composition subsequently had no effect on bee visitation rate. Yet we observed that plants with higher female nectar volumes were more frequently visited by bumble bees and resulted in higher fruit set. Female flowers produce substantially more nectar than male flowers suggesting high volume is a primary mechanism for attracting pollinators. Additionally, higher nectar volumes could also increase bee handling time and likelihood of pollen deposition^46^. Interestingly, the number of fruit per plant was positively related to male flower visitation rate (Fig. 4; Supplementary Table S5), potentially because female flower visitation and pollen transfer was more likely on these plants because of the male traits explored above, and they had more flowers (Fig. 4). Therefore, maintaining nectar composition, concentration, and volume is a key ecological trait for pollinator attraction and pollination success that seemed to be buffered in the face of stressful conditions. We observed that all plants produced fruit, and therefore maintaining reward qualities of male and female flowers under a variety of soil conditions may perhaps insure pollinator attraction in stressful environmental conditions.

There are some limitations and new perspectives to our study which could be explored in future research. First, nitrogen and phosphorus treatments were coupled in our study. The highest level of nitrogen was paired with our middle phosphorus treatment at an 8:1 nitrogen:phosphorus ratio; perhaps the negative effect observed at high nitrogen concentrations could have been mitigated by higher phosphorus levels. Further research on the effects of one nutrient independent of one another and across a wider range of nutrient concentrations and ratios would help understand the asymptotic or potential detrimental effect of higher nutrient concentrations of both nitrogen and phosphorus. Second, there may be other detrimental effects on reproductive quality that we did not measure. These include reduced seed number per fruit by reduced pollinator visitation^47–51^ and reduced seed viability from soil nutrients either in seed development or for seed germination^52,53^. Third, we studied cucumbers which have undergone extensive artificial selection for particular traits^54^. Perennial plants, which are less reliant on a single season for reproduction, or non-cultivated plants may exemplify higher plasticity than annual plants^55^. Therefore, similar studies should be conducted with wild populations of plants and with different pollination systems. Finally, it is important to study the interaction between plants’ floral responses to changes in soil nutrients and important factors related to climate change (drought, temperature, CO_2_) and how these scale up to pollinator attraction and plant reproduction^18,19,38,56^.

In this study we present the effects of multiple soil nutrient titers on the entire plant life cycle, with particular attention drawn to the effects of nutrients on floral display and rewards and pollinator attraction and visitation, and finally reproductive success. Nutrients clearly can affect pollination success of annual plants, but these plants may be somewhat buffered through particular consistent or fixed floral traits. However, because this study focused on a single plant species, future assessments of the responses on a diversity of wild and agricultural populations of plants, and their associated pollinator communities, to nutritional stress caused by anthropogenic activity are critical. Additionally, the impacts of soil nutrition-induced changes in plant nutritional rewards on pollinator health should be considered. With the effects of anthropogenic activities likely to increase in scope and scale, evaluating the full extent of the impacts on natural and agricultural ecosystems is essential.

## Methods

### Plant rearing and fertilizer treatments

We used Marketmore 76 cucumbers, a monoecious variety (separate male and female flowers grow on the same plant) that are non-parthenogenetic, requiring insect pollination to set seed and fruit^54^. The cucumbers were unrestricted commercially bought non-transgenic seeds (Johnny’s Selected Seeds, Waterville, ME); no permit was needed for use and the research followed relevant institutional and national guidelines and legislation. All cucumbers were grown at the Penn State College of Agricultural Sciences greenhouses under the same conditions. Day length was scheduled for 14 h at 24–28 °C and 74–80% humidity under full spectrum LED grow lights by day and 20–25 °C and 68–78% humidity by night. All pots were washed and sterilized with steam. We planted and germinated sets of three seeds in starter pots with Sunshine #4 potting mix until the 2nd true-leaf stage. At 21 days from planting, we repotted one of each set of three seedlings to its own 3 gallon pot with Sunshine #4 potting mix (composed of perlite, peat moss, and silicon, containing insignificant nitrogen or phosphorus nutritional value). We built a trellis for each individual plant using bamboo stakes. There were 15 plants per treatment group, each group on a separate table. For the duration of the study, for each treatment group, we watered each plant using a drip irrigation system from a 60 L barrel with an electronic pump with a drip line sent to each pot. We watered each individual plant with its associated treatment fertilizer solution 1x/day until water flowed from the bottom of the pots (soil moisture was not measured because all plants were watered under the same regime).

There were five fertilizer solutions (a control and four treatments), with different nitrogen and phosphorus concentrations and ratios, which were mixed into each barrel (Supplementary Table S1). Our control added no additional nitrogen or phosphorus, hereby considered 0 ppm nitrogen and phosphorus. Treatment 1 contained 50 ppm nitrogen and 25 ppm phosphorus at a ratio of 2:1 nitrogen:phosphorus (N:P); treatment 2 contained 100 ppm nitrogen and 25 ppm phosphorus at 4:1 N:P; treatment 3 contained 200 ppm nitrogen and 25 ppm phosphorus at 8:1 N:P; treatment 4 contained 200 ppm nitrogen and 50 ppm phosphorus at 4:1 N:P. Our solutions used tap water and we also added additional minerals for pH buffering.

### Bee pollinator rearing

Two Biobest (Biobest Canada Ltd., Leamington, ON) bumble bee, *Bombus impatiens* C. (Hymenoptera: Apidae), research colonies with approximately 50–75 workers and one queen were used to evaluate bee visitation rates. The bees were fed honey bee collected pollen and Biobest proprietary sugar water *ad libitum* until used for visitation observations to the cucumbers. We housed the colonies in a separate greenhouse bay from the cucumber plants (under the same environmental conditions as the cucumbers), and moved plants to this room during the foraging assays (see below). The use of *B. impatiens* does not require ethical approval for research purposes.

### Data collection

#### Vegetative traits

For each individual plant, we measured plant height from the surface of the soil on the 55th day of growth after repotting when the fruits of the plants began developing. We measured above ground dry biomass on the 62nd day of growth after repotting (when plants stopped growing in height) after drying the plant at 65 °C for 48 h.

#### Flower production and floral display

To measure flower production and display, we recorded the number of days from seeding until first bloom. We determined the total number of flowers per plant by counting the number of open male and female flowers once per day every day at 0900 from the 33rd day of growth after repotting (when budding was first observed) until the 61st day. Counted flowers were marked with strings so that only new flowers were counted every day. We determined average flower size per plant by measuring the corolla width (cm) of five male and five female flowers.

#### Nectar volume, concentration, and composition

To measure nectar rewards, we measured the average nectar volume of five mature male and five female flowers per plant using 2 µl microcapillary tubes. The same sample was used to measure average nectar concentration with a refractometer (Eclipse, Stanley and Bellingham, Basingstoke, UK;^57^).

To measure nectar composition, in a separate sample, we used pooled nectar obtained from five male and five female flowers per plant to measure male and female sucrose, glucose, and fructose proportions. We placed the entirety of the samples on filter paper and stored them at -20 °C until analysis. We soaked the filter paper in 1.2 ml H_2_O, evaporated the sample in a speed vac, then resuspended in water at 1:100 of the original volume of the sample. The sample was then filtered in a 0.45 µm microspin centrifuge filter (13500 RCF for 5 m) to remove any particles. We determined fructose, glucose, and sucrose concentrations using a Roche Diagnostics “Yellow line” Sucrose/D-Glucose/D-Fructose kit (R-Biopharm # 10,716,260,035, Washington, MO). The protocol was modified for analysis using 300 µl 96 well plates. Sample absorbance values were measured using a SpectraMax 190 spectrophotometer (Molecular Devices, San Jose, CA). Samples were blocked by treatment, sex, and plant across plates and replicated 3 times. To ensure accurate results, plate means were compared using a two-way ANOVA (treatment × plate), grouped by sugar type and flower sex (Female: Sucrose df = 17, *F* = 0.58, *P* = 0.89; Glucose df = 17, *F* = 1.12, *P* = 0.36; Fructose df = 17, *F* = 1.06, *P* = 0.42; Male: Sucrose df = 18, *F* = 0.97, *P* = 0.51; Glucose df = 18, *F* = 1.10, *P* = 0.38; Fructose df = 18, *F* = 1.06, *P* = 0.42). Each plate was run with serial dilutions of analytical grade D-fructose, D-glucose, and sucrose. Results were also corroborated with HPLC analysis. Sugar concentrations were calculated as w/w then converted to proportions of total sugar.

#### Pollen quantity and quality

To determine the average number of pollen grains of three male flowers per plant, we removed the anthers and dried the pollen on wax paper for 48 h at room temperature. We transferred the pollen to a solution of aniline-blue in lactophenol and mounted the pollen on a slide. We photographed each slide under a dissecting microscope, converted each image into a binary image, then counted pollen grains using the “analyze particles” function in ImageJ^58–60^.

We pooled pollen in each treatment group for nutritional analysis. Using different synthetic paint brushes for each treatment group, we brushed pollen from dehisced anthers evenly across all plants until we obtained > 10 mg pollen from each treatment group. The pollen was analyzed for its protein, lipid, and sugar concentrations as µg nutrient/mg pollen, and P:L macronutrient ratios^23,24^.

#### Pollinator visitation

Pollinator visitation rates were monitored for 10 days. Each day we randomly arranged three individual plants per treatment in a grid in a separate greenhouse room where the two bumble bee colonies were housed. We opened the colonies at 10:00 AM so the bees could forage freely and after they emerged and oriented (~ 5–10 min) we began observations. On each day of observations, we observed each individual plant for 1 min and counted the number of bees visiting and collecting pollen or nectar from male and female flowers and repeated this for six rounds^61^. In total, we collected visitation data for each individual plant on two separate days. We calculated the visitation rate as the number of male or female flower visits per minute divided by the number of male or female flowers per plant.

#### Fruit quality, number, and set

We counted the total number of mature cucumbers on the 62nd day after repotting as a measure of total fruit number per plant. We also determined the fruit set of each plant as the percentage of total fruit per total number of female flowers per plant. Finally, we determined the total dry weight of fruit by first randomly selecting five fruit per plant (or all if  ≤ 5/plant) and drying them at 65 °C for 72 h. To determine total dry weight of fruit per plant (W), we measured fresh mass of all fruit (W1) and multiplied that by the dry mass (W3) divided by the fresh weight (W2) of the fruit selected (W = (W3/W2)*W1).

### Analysis

All data used for analysis are provided in Supplementary Information Datasets.

#### Regression

We analyzed the data to determine the main effects of soil nutrients on vegetative traits, floral display, and floral rewards. We (1) determined the treatment effects of nitrogen and phosphorus ppm (nitrogen = 0, 50, 100, 200 ppm; phosphorus = 0, 25, 50 ppm), and the nitrogen × phosphorus interaction, or (2) the effects of nitrogen:phosphorus ratios (N:P ratios = 1:1, 2:1, 4:1, 8:1) as continuous variables. We ran multiple linear regression analyses of nutrient treatment on the following traits: plant height (m), plant dry biomass (g), days from seed to first flower (d), number of total male and female flowers, total male to female flower ratios (m:f ratio), male and female flower corolla size (cm), male and female nectar volume (µl) and concentration (%), male and female nectar sucrose, fructose, and glucose composition (% of total sugar), pollen number (× 10^3^), pollen protein (µg/mg), and lipid content (µg/ml), pollen protein to lipid ratios (P:L), and average fruit dry mass per plant (g). Because nitrogen and phosphorus concentrations were coupled in the treatment groups, we ran a multiple regression with nitrogen and phosphorus and nitrogen × phosphorus interaction as fixed effects, using a false discovery rate (FDR) and Adjusted *R*^2^ analysis to verify significance. To further explore the interaction of soil nutrient ratios on plant growth and floral traits, the regression was fitted with linear and polynomial terms of nitrogen:phosphorus ratios as fixed effects on the same variables as above.

We then used multiple linear regression to analyze the effects of plant and floral traits on visitation rates to male and female flowers separately. Our fixed effects were plant height, days to first flower, the number of male and female flowers, m:f flower ratios, male and female flower size, nectar volume, and concentration, and pollen number per flower. Finally, we used linear regression to test if male and female visitation rate affected fruit number, fruit set, and fruit dry mass. All regression analyses were conducted in JMP®, Version 15 (SAS Institute Inc., Cary, NC, 1989–2021).

#### Piecewise structural equation modeling

To consider plant life cycle from growth to pollinator attraction and pollination to fruit production, we used structural equation modeling to determine the best path and effect sizes from soil nutrients to fruit production. We used the *piecewiseSEM* package in R^39^ in two separate models of female vs. male fitness (Fig. 5, Supplementary Table S6, S7). Linear regressions were built for the following growth stages: vegetative traits and floral traits ~ soil nutrients; pollinator attraction/visitation ~ floral traits; yield/reproduction ~ pollinator visitation (Fig. 5, S2, Supplementary Table S6, 7 for model details). We conducted these paths separately to include either only nitrogen and phosphorus as independent variables alone or including their interaction. Using model selection via AIC, AICc, and BIC, and choosing relationships determined to be significant (but logical in terms of plant growth) via “d-separation” tests, we report the “best” male and female fitness models in the results.

## Supplementary Information

Supplementary Figure S1.

Supplementary Figure S2.

Supplementary Tables.

Supplementary Information.

## Data Availability

All data generated or analyzed during this study are included in this published article (and its Supplementary Information files).
