# Supplementary material for: Impacts of soil nutrition on floral traits, pollinator attraction, and fitness in cucumbers (Cucumis sativus L.)
Source: Sci Rep. 2022 Dec 16;12:21802. doi: 10.1038/s41598-022-26164-4 (PMC9758155; doi:10.1038/s41598-022-26164-4)
Supplement: Supplementary file 1 — Supplementary Figure S1. [file 41598_2022_26164_MOESM1_ESM.pdf]

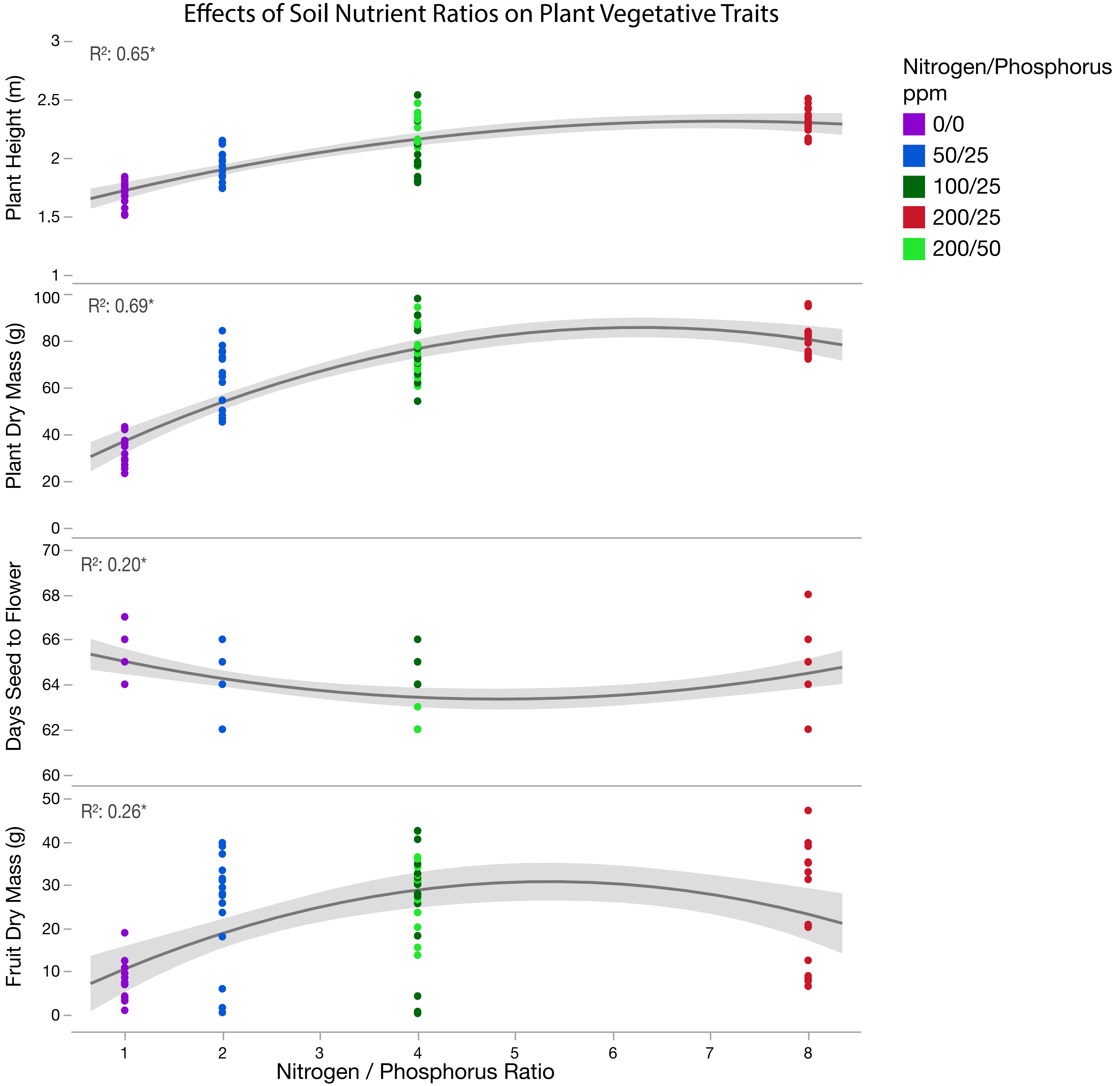

Supplementary Figure S1. The effects of nitrogen:phosphorus soil nutrient ratios on plant and fruit vegetative traits (Supplementary Table S3). Regression lines are fitted with the quadratic ratio terms. Each marker represents a different individual plant, and each marker is colored by their treatment group. Nitrogen ppm/phosphorus ppm of nutrient solutions for each treatment group are labeled in the legend. Note that plant traits improve as ratios increase to 4:1, yet reach asymptotic levels at 8:1. The effects of nitrogen and phosphorus individually are provided in Fig. 5, Fig. S2, and Supplementary Table S2.
