# Supplementary material for: Impacts of soil nutrition on floral traits, pollinator attraction, and fitness in cucumbers (Cucumis sativus L.)
Source: Sci Rep. 2022 Dec 16;12:21802. doi: 10.1038/s41598-022-26164-4 (PMC9758155; doi:10.1038/s41598-022-26164-4)
Supplement: Supplementary file 2 — Supplementary Figure S2. [file 41598_2022_26164_MOESM2_ESM.pdf]

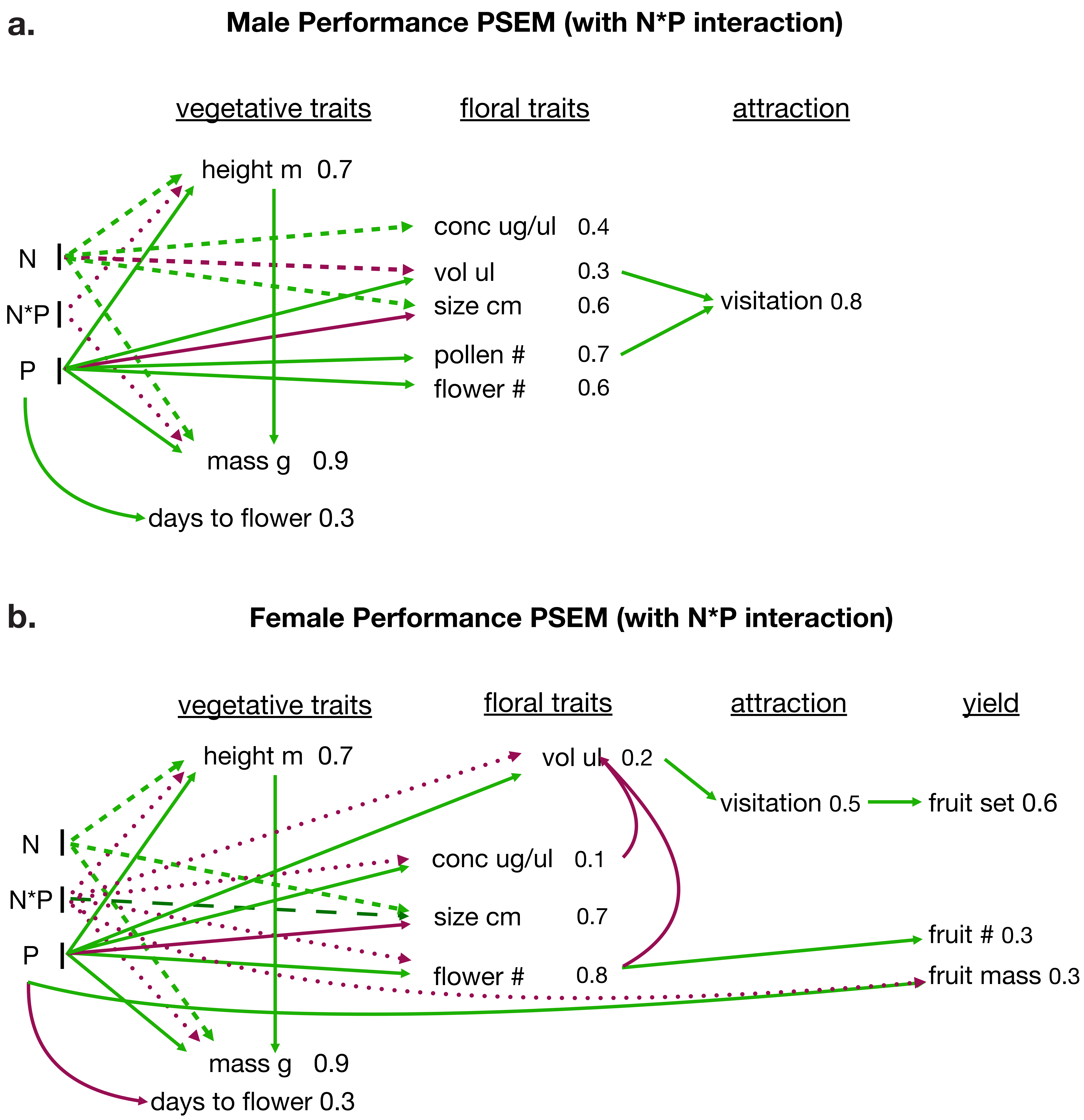

Supplementary Figure S2. Piecewise SEM path analyses of soil nutrients on vegetative traits and floral traits, pollinator attraction/visitation, and reproduction/yield measures for male and female traits in cucumbers. These models differ from Fig. 5 in that they include soil nitrogen×phosphorus ppm interaction. Green lines represent significant positive relationships, whilst red lines represent significant negative relationships. Nonsignificant relationships are not represented for ease of path interpretation (full model paths and details are provided in Supplementary Tables S6,S7). R2 values for each linear regression response variable are provided. Estimated effects (including standardized) for each variable are provided in Supplementary Tables S6,S7. Note that including the nitrogen×phosphorus interaction may have resulted in more complex predictions of floral traits (and possibly overfitting). Overall model results remained consistent with Fig. 5. Note however, the complex interacting influences predicting female nectar volume that were not present in any other models tested in the study, representing the difficulty in determining female nectar volume, and how it remained consistent across treatment groups.
